# Assessment of Vitamin C and Antioxidant Profiles in Saliva and Serum in Patients with Periodontitis and Ischemic Heart Disease

**DOI:** 10.3390/nu11122956

**Published:** 2019-12-04

**Authors:** Gaetano Isola, Alessandro Polizzi, Simone Muraglie, Rosalia Leonardi, Antonino Lo Giudice

**Affiliations:** 1 Department of General Surgery and Surgical-Medical Specialties, School of Dentistry, University of Catania, Via S. Sofia 78, 95124 Catania, Italysimonemuraglie@live.it (S.M.); rleonard@unict.it (R.L.); nino.logiudice@gmail.com (A.L.G.); 2 Department of Biomedical and Dental Sciences and Morphofunctional Imaging, School of Dentistry, University of Messina, 98125 Messina, Italy

**Keywords:** vitamin C, retinol, α-carotene, β-carotene, β-cryptoxanthin, γ-tocopherol, lutein, zeaxanthin, lycopene, periodontitis, ischemic heart disease, C-reactive protein, cardiovascular disease, clinical trial

## Abstract

Vitamin C and antioxidants play a crucial role in endothelial function and may be a link for the known interaction of periodontitis and ischemic heart disease (CAD). This pilot study evaluates the association of gingival health, periodontitis, CAD, or both conditions with salivary and serum vitamin C and antioxidant levels. The clinical and periodontal characteristics, serum, and saliva samples were collected from 36 patients with periodontitis, 35 patients with CAD, 36 patients with periodontitis plus CAD, and 36 healthy controls. Levels of vitamin C, antioxidants, and C-reactive protein (hs-CRP) were assessed with a commercially available kit. The median concentrations of salivary and serum vitamin C and antioxidants (α-tocopherol, β-carotene, lutein, and lycopene) were significantly lower in the CAD group (*p* < 0.001) and in the periodontitis plus CAD group (*p* < 0.001) compared to periodontitis patients and controls. In univariate models, periodontitis (*p* = 0.034), CAD (*p* < 0.001), and hs-CRP (*p* < 0.001) were significantly negatively associated with serum vitamin C; whereas, in a multivariate model, only hs-CRP remained a significant predictor of serum vitamin C (*p* < 0.001). In a multivariate model, the significant predictors of salivary vitamin C levels were triglycerides (*p* = 0.028) and hs-CRP (*p* < 0.001). Patients with CAD and periodontitis plus CAD presented lower levels of salivary and serum vitamin C compared to healthy subjects and periodontitis patients. hs-CRP was a significant predictor of decreased salivary and serum vitamin C levels.

## 1. Introduction

About 50% of adults in the United States of America (USA), aged over 30 years, are affected by periodontitis, and almost 10% of the world population have a severe form of periodontal disease [1,2]. Periodontitis can be defined as a chronic inflammatory multifactorial disease caused by periodontal bacteria that determine the destruction of the tooth-supporting tissues, including alveolar bone, and which can lead to tooth loss [3]. Some observational studies during the last few decades have shown a direct and positive association between periodontitis and coronary heart disease, known also as ischemic heart disease (CAD), including myocardial infarction, stroke, and cardiovascular disease (CVD) [4,5]. More specifically, recent large cohort studies and a systematic review highlighted a positive graded association between periodontitis and increased risk of stroke and CAD [6,7,8].

The etiology of periodontitis comprises inflammatory and immunological processes that cause dysregulation in the host response due to the superinfection of periodontal bacteria [9]. Moreover, periodontitis has been positively associated with higher serum levels of different inflammatory biomarkers, such as interleukin 6 (IL-6), IL-17, C-reactive protein, and prostaglandins [10].

Vitamin C has been shown, together with some other antioxidant agents, to be an endogenous modulator of the metabolism of nitric oxide (NO) and subsequent endothelium-dependent vasodilation [11]. NO is one of the important mediators that regulate function and vasodilatation of the endothelium, because it controls the level of inflammation in the vessels, vascular tone, and cell proliferation, and it modulates the release of different growth factors [12]. Vitamin C has been extensively used to evaluate and predict early signs of endothelial dysfunction and CAD events [13]. A prospective study on 200 patients with heart failure showed that patients with high serum vitamin C deficiency moderated the relationship between inflammation and CAD events [14]. Furthermore, a multi-trial study on 134 subjects with CAD showed a therapeutic use of vitamins C and E against the reperfusion damage produced during angioplasty [15].

Few reports have associated periodontitis with CAD, endothelial dysfunction, and augmented the risk of CVD [16,17,18]. It has previously been hypothesized that several inflammatory mediators are systemically released during periodontitis, including CRP, metalloproteases, and prostaglandins, into the bloodstream and decrease the production of NO [19]. The reduced production of NO negatively impacts the vascular endothelial cells, whose impairment determines, finally, endothelial dysfunction, vasodilatation, and CAD [20,21]. Hence, this has aroused interest in assessing possible oral factors that influence and regulate endothelial changes as subclinical signs of CAD.

Previous studies have demonstrated an indirect association between high serum vitamin C and a direct association between high CRP levels and consequent endothelial damage in patients with periodontitis [22,23].

The local production of NO has an essential role in the development and progression of periodontitis. Both increases and decreases in the production of salivary NO metabolites during periodontitis in gingival tissue against periodontal bacteria and periodontal tissues have been reported to be associated with impaired endothelium-dependent vasodilatation [24]. More specifically, it has been shown that vitamin C and several antioxidants act as a competitive stimulator of the NO synthase, and that lower serum vitamin C levels have been reported in several metabolic disorders, including periodontitis [25,26].

All of these studies were performed only on serum vitamin C. To date, few studies have evaluated salivary vitamin C levels during periodontitis. Moreover, there are insufficient data on the association of periodontitis on both serum and salivary vitamin C levels during periodontitis and CAD.

The aims of this study were to evaluate a possible association of gingival health, periodontitis, CAD, or a combination of both diseases on saliva and serum vitamin C and antioxidant levels. Moreover, the association between both saliva and serum vitamin C levels were assessed, and whether salivary or serum vitamin C levels were mediated by serum CRP in patients with periodontitis and with CAD.

## 2. Materials and Methods

### 2.1. Study Design

The study population consisted of 309 patients with periodontitis, CAD, and healthy controls selected among those who attended the Department of Periodontology, School of Dentistry, from June 2016 to October 2018. Groups were selected from a prespecified age range (40–60) and sex so that a similar proportion to the cases fall into the categories defined by the selection variable (sex and age in this study). Fifty percent of the cases and controls were males aged 45–58 years.

The study was performed by the Declaration of Helsinki, revised in 2016 by medical research. Ethical approval was obtained from the local IRB of the University of Messina (012-2016). The study was registered at clinicaltrials.gov (NCT03873789). Written informed consent was obtained from each patient about the study characteristics and possible risks of the study. This study followed the STROBE guidelines for the strengthening of reporting of observational studies (Table S1) [27].

Inclusion criteria for the periodontitis group were: (1) Presence of at least 16 teeth; (2) a minimum of 40% of sites with clinical attachment level (CAL) ≥2 mm and probing depth (PD) ≥4 mm [28]; (3) presence of at least one site for each quadrant with ≥2 mm of crestal alveolar bone loss verified on digital periapical radiographs; and (4) presence of ≥40% sites with bleeding on probing (BOP) [29]. Healthy individuals presented no systemic disease, ≤10% sites with BOP, no sites with PD ≥4 mm or CAL ≥4 mm, no sites with BOP [29] or radiographic signs of bone loss.

Inclusion criteria for the CAD group were: At least ≥18 years old with a diagnosis of CVD, ≥50% of stenosis of at least one coronary artery verified by coronary angiography or a coronary artery bypass surgery, or past or current percutaneous coronary intervention [30]. Moreover, information on previous medical conditions, cardiovascular risk factors, medications, electrocardiography, echocardiography, and coronary angiogram results were collected. The inclusion criteria for the periodontitis + CAD group were based on the same criteria of the single periodontitis and CAD groups but combined.

The exclusion criteria for all patients were: (1) Use of contraceptives; (2) use of antibiotics, immunosuppressive or anti-inflammatory drugs throughout the last three months prior to the study; (3) status of pregnancy or lactation; (4) previous history of excessive drinking; (5) allergy to local anaesthetic; (6) use of drugs that may potentially determine gingival hyperplasia such as Hydantoin, Nifedipine, Cyclosporin A, or similar drugs; (7) periodontal therapy throughout the last three months prior to the study.

**Figure 1:**
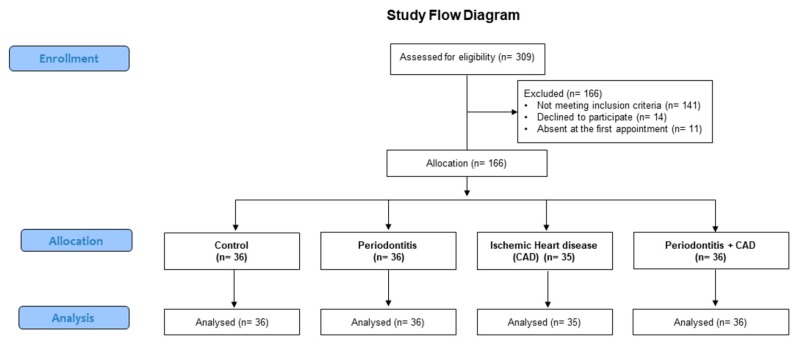
Flowchart of the study.

After a first screening, 166 patients were excluded from the final sample because they did not meet the inclusion criteria (*n* = 141), declined to participate (*n* = 14), or did not attend the first appointment (*n* = 11). Finally, for this study, 36 patients with periodontitis, 35 patients with CAD, 36 patients with periodontitis plus CAD, and 36 healthy subjects were finally enrolled (Figure 1).

The demographic (level of education), clinical and medical characteristics (sex, age, body mass index, hypertension, diabetes, dyslipidemia, previous CVD events), and medications were assessed in all enrolled subjects. The presence of diabetes mellitus was based on the history of the patient or a fasting blood glucose ≥126 mg/dL. Body Mass Index (BMI) was estimated on the weight of the patient divided by the square of the patient’s height, i.e., kilogram per square meter (kg/m^2^).

The periodontal evaluation comprised probing depth (PD), clinical attachment loss (CAL), bleeding on probing (BOP), and plaque score (PI) [31], and the presence of bleeding was recorded up to 30 s after probing. CAL was recorded as PD plus recession, with the cementoenamel junction as a reference for CAL measurements. All clinical periodontal parameters were recorded, in all patients, at six sites per tooth on all teeth present, excluding third molars, by two independent calibrated examiners (a principal examiner and a second control examiner) not involved in the subsequent data analysis with a manual periodontal probe (UNC-15, Hu-Friedy, Chicago, IL, USA). The inter- and intra-examiner reliability of the outcomes PD and CAL were assessed using the intraclass correlation coefficient (ICC). The inter-examiner reliability resulted in an agreement for PD (ICC = 0.817) and CAL (ICC = 0.826), denoting a reasonable degree of reliability for both parameters. The intra-examiner reliability of PD and CAL was performed only on 20 selected patients (five patients per group chosen randomly) for both examiners. The intra-examiner reliability for the first examiner resulted in an agreement for PD (ICC = 0.834) and CAL (ICC = 0.809), and for the second examiner, it resulted in an agreement for PD (ICC = 0.851) and CAL (ICC = 0.819), denoting a reasonable degree of reliability for both parameters.

A power analysis was performed to calculate the minimum sample size required. The sample size was established considering a number of groups equal to 4, an effect size of 0.30 for vitamin C (that represented the primary outcome variable), an expected standard deviation of 1.5 [25], a 2-sided significance level of 0.05, and a power of 80%. It was determined that approximately 32 patients per group would be needed. Thus, it was estimated that 128 subjects were needed to ensure a power level of 80%. One hundred and forty-three patients were enrolled so that the study achieved a power of 83%. Power and sample size calculations were performed using statistical software (G*Power version 3.1.9.4, Universitat Dusseldorf, Germany).

### 2.2. Vitamin C Assessment in Saliva and Serum

Fasting samples were collected in all subjects between 8:00 and 10:00 am. Participants were asked to refrain from eating, drinking, chewing gum, brushing teeth, as well from using any mouthwashes, in the last 12 h before the sampling.

The venous puncture was performed, and blood samples were collected, cooled on ice immediately, and centrifuged at 4 °C (800× *g* per 10 min). Serum samples were stabilized immediately using metaphosphoric acid in order to avoid oxidization of vitamin C. To collect saliva, subjects were asked to chew on a cotton roll for 2 min, and saliva samples were collected using Salivette collection devices (Sarsted, Verona, Italy) and immediately centrifuged at 4 °C (1000× *g* per 2 min). Serum and saliva samples were stored at −20 °C until analysis.

Levels of vitamin C and antioxidants (retinol, α-carotene, β-carotene, γ-tocopherol, β-cryptoxanthin, lutein, zeaxanthin, and lycopene) were assessed by use of the commercially available kit for high-performance liquid chromatography (HPLC) measurements (Eureka, Ancona, Italy). In fasting conditions, levels of C-reactive protein (hs-CRP) were assessed by a commercially available nephelometric assay. An hs-CRP level higher than 3 mg/L was associated with an increased risk of CAD. Plasma lipids and glucose were determined by routine methods.

### 2.3. Statistical Analysis

The numerical data are expressed as median, 25% and 75% percentiles, and categorical variables as number and percentage. The Kruskal–Wallis test was applied in order to compare the four groups with regard to all numerical variables, and the Mann–Whitney test in order to perform two-by-two comparisons between groups. Since most of the examined variables (e.g., triglycerides, fasting glucose, and all periodontal variables) did not present normal distribution, as verified by a Kolmogorov–Smirnov test, the analysis was performed by non-parametric tests. For these multiple comparisons, Bonferroni’s correction was applied, for which the significant alpha level 0.050 was divided by the number of possible comparisons (*n* = 6), so the “adjusted” significance level for this analysis was equal to 0.050/6 = 0.008. A *p*-trend was performed with the Jonckheere–Terpstra Test for serum and salivary vitamin C levels to assess whether the vitamin C levels were significantly increased in healthy, periodontitis, CAD, and periodontitis + CAD patients. The Spearman correlation test was applied to determine the existence of any significant interdependence between hs-CRP, serum, and salivary vitamin C.

In all enrolled subjects, univariate and multivariable linear regression models were performed in order to assess the dependence of salivary and serum vitamin C levels on potentially explicative variables such as age, gender, education, socioeconomic status (SES), BMI, CRP, triglycerides, total cholesterol, and antioxidants. In the final multivariate model, only age, gender, education, and SES were included as confounders, and tests were carried out to analyze if periodontitis, CAD, and hs-CRP influenced serum vitamin C levels. The same analysis was performed for salivary vitamin C as an outcome. Statistical analyses were performed using statistical software (SPSS 22.0 for Windows package (SPS Srl, Bologna, Italy)). A *p*-value < 0.05 was considered statistically significant.

## 3. Results

The patient characteristics and biochemical parameters of the recruited subjects are summarized in Table 1. Controls and patients were matched for age and gender, and there were no significant differences between the distribution of education levels or median values (25% and 75% percentiles) of BMI, triglycerides, or total cholesterol between the groups (Table 1). Increased values of hs-CRP were observed among patients with periodontitis, CAD, and periodontitis + CAD in comparison with healthy subjects (*p* < 0.001). Patients with CAD and periodontitis + CAD had a similar proportion of previous CVD events (atrial fibrillation, angina pectoris, stroke, heart failure) and took more CVD drugs (antihypertensive, statins, low-dose aspirin, beta-blockers). Patients with CAD and periodontitis + CAD presented lower serum retinol, α-carotene, β-carotene, γ-tocopherol, β-cryptoxanthin, lutein, zeaxanthin, and lycopene levels compared to periodontitis and healthy controls (Table 1).

Table 2 shows dental variables in patients with periodontitis, CAD, periodontitis + CAD, and controls. Patients with periodontitis and with periodontitis + CAD presented a lower median number of teeth and higher median values of periodontal parameters (CAL, PD, BOP, PI) compared with CAD and control subjects (*p* < 0.001). Moreover, the median values of periodontal parameters were significantly higher in patients in the periodontitis and periodontitis + CAD groups compared to patients with CAD and healthy controls (*p* < 0.001, Kruskal–Wallis test) (Table 2).

### Vitamin C Evaluation

**Figure 2:**
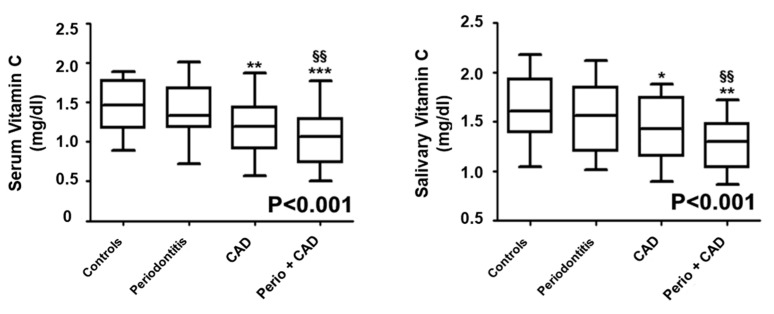
Median values (25% and 75% percentiles) of serum and salivary vitamin C levels in each group of subjects. * *p* < 0.05, ** *p* < 0.01, and *** *p* < 0.001 significant differences vs. control subjects (derived by Kruskal–Wallis test). ^§§^ *p* < 0.01 significant differences vs. periodontitis patients. *p* < 0.001 (obtained by Jonckheere–Terpstra test).

Median (25th and 75th percentile) serum and salivary vitamin C levels are presented in Figure 2. The median concentrations of serum and salivary vitamin C were lower in the CAD (*p* < 0.01) and in the periodontitis + CAD (*p* < 0.001) groups compared to controls. Serum and salivary vitamin C concentrations were also significantly decreased in patients of the periodontitis + CAD group in comparison with periodontitis patients (*p* < 0.01; Figure 2). Overall, the *p*-value for trend analysis performed (Jonckheere–Terpstra test) indicated that serum vitamin C progressively decreased in patients with periodontitis, CAD, and periodontitis + CAD (*p* < 0.001; Figure 2).

**Figure 3:**
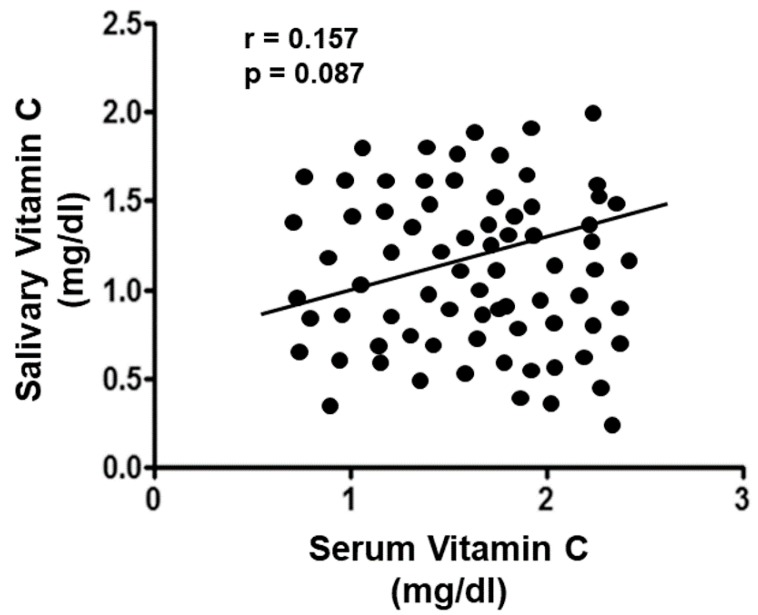
Correlation analysis of serum and salivary vitamin C levels in all enrolled subjects.

There was no statistically significant correlation between salivary and serum vitamin C levels (rs = 0.157, *p* = 0.087; Figure 3).

**Figure 4:**
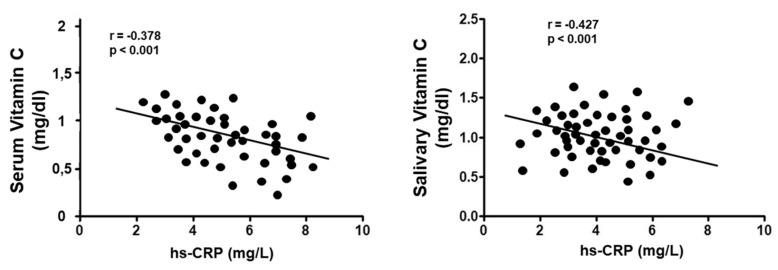
Correlation analysis of serum and salivary vitamin C levels with CRP values in all enrolled subjects.

Moreover, across all subjects, serum/salivary vitamin C concentrations correlated negatively (rs = –0.378, *p* < 0.001)/(rs = −0.427, *p* < 0.001) with hs-CRP levels (Figure 4).

The adjusted multivariate linear regression analysis, aimed at assessing the possible association of periodontitis and CAD on serum and salivary vitamin C levels, showed that hs-CRP (*p* < 0.001) was the only statistically significant predictor variable for serum vitamin C; hs-CRP (*p* < 0.001) and triglycerides (*p* = 0.028) were the statistically significant predictor variables for salivary vitamin C (Table 3).

## 4. Discussion

This study evaluated the association of different conditions such as gingival health, periodontitis, CAD, or a combination of both diseases (periodontitis and CAD) on saliva and serum vitamin C levels. This study found that periodontitis in CAD patients was associated with decreased levels of serum and salivary vitamin C and hs-CRP levels. However, compared to periodontitis and healthy subjects, only patients with CAD and periodontitis + CAD presented significantly lower salivary and serum vitamin C levels, supporting the hypothesis that CAD may have contributed to decreased serum and salivary vitamin C levels.

Moreover, our results showed that the presence of periodontitis in patients with CAD might serve as an inhibitor of vitamin C and for associated risk of CAD and CVD. Recent investigations suggested that low serum vitamin C levels, through inactivation of NO signaling, are independent risk factors of CVD and related to increased mortality [32]. More specifically, it has also been demonstrated that a decrease in vitamin C levels was associated with carotid endothelial damage in patients with atherosclerosis, highlighting the positive role of vitamin C and antioxidants on NO levels [33]. The co-occurrence of periodontitis in CAD patients may be a possible pathway for the observed deterioration of endothelial function via decreased vitamin C levels. Periodontal treatment clinically decreased serum vitamin C and antioxidants levels in patients with chronic kidney disease [34].

As a support of the present study, several lines of evidence have shown that stimulating oxidative stress conditions, such as periodontitis and CAD, may have led to the lower the production of vitamin C, which in turn could augment serum and salivary CRP levels [35]. The high inflammation present during periodontitis and CAD is believed to accelerate vitamin C oxidation [35]. In accordance with our results, Amaliya et al. [36] found that low serum vitamin C and high CRP levels were associated in a dose-dependent manner in a sample of 98 subjects with periodontitis.

Moreover, while there are some observations on the serum vitamin C levels as a marker for endothelial dysfunction or CAD risk, there are no reports that analyze both salivary and serum vitamin C levels during periodontitis. However, the present study did not find a statistically significant correlation between serum and salivary vitamin C levels; salivary vitamin C levels were associated by hs-CRP levels. This could be explained by the fact that the saliva levels of vitamin C could mainly reflect the serum vitamin C levels or that the salivary vitamin C levels may have been influenced by the saliva collection method used in the present study [37].

In the present study, patients with CAD and with periodontitis plus CAD presented low salivary vitamin C levels, in accordance with previous studies which demonstrated that saliva contains many biochemical systems known to be involved in soft-tissue repair, and many antibacterial components, including lysozyme, lactoferrin, and salivary peroxidase [38]. Human whole saliva contains a complex peroxidase system, the major components of which include different forms of lactoperoxidase secreted by the salivary glands and myeloperoxidases from polymorphonucleocytes [39].

While the systemic impact of reduced vitamin C levels on endothelial dysfunction via decreased NO has been demonstrated, the effect of oral vitamin C is less clear. As a matter of fact, there are reports which show that periodontitis is positively associated with impaired salivary NO levels [19,24]. NO can be produced in the gingival tissues as part of the oral unspecific salivary antibacterial defense against anaerobic periodontopathogens bacteria [12,24,40]. In this regard, some reports showed high levels of NO synthesis and activity in the inflamed periodontal tissue [25,41,42,43,44]. Another explanation for the contradictory results may be due to the method of saliva collection. Moreover, it can also be argued that the difference in NO production at the periodontal level is probably different from NO in the bloodstream: In the mouth, it is an antibacterial defense, whereas systemically, it impacts endothelial function.

Moreover, endothelial dysfunctions in periodontitis patients with CAD could be due to a specific immunoreactive pathway in which vitamin C modulates an anti-inflammatory response against periodontopathic bacteria during periodontitis. It has been shown that vitamin C, during periodontitis is involved in immune response through the activated endothelium and its heat shock proteins that are present in the endothelium surface, finally stimulating some cross-reactive T-cells with particularity for host-activated antibodies [45,46]. This process, modulated by vitamin C, also affects the inducted defense mechanism mediated by NO, which promotes the hyperactivation of the endothelial cells that increases the risk of further infection or systemic inflammation due to periodontitis [47,48,49].

However, the present preliminary study presents some limitations. One of the main limitations is the cross-sectional nature of the study, which does not allow any evaluation on the impact of vitamin C levels on periodontitis, which should be assessed only with a longitudinal observation. Another limitation is the small sample size, which was due to matching age, gender, and education. An advantage of matching is the elimination of the impact of these confounding variables. Significant limitations also include the lack of analysis for dietary quality (e.g., intake of vitamin C and statins) and the analysis of alpha-tocopherol.

## 5. Conclusions

During the last few decades, new approaches through salivary diagnostics have been developed to evaluate the possible useful biomarkers for predicting the disease. This study indicated that patients who have periodontitis and CAD presented lower serum and salivary vitamin C and antioxidant levels compared to patients with periodontitis and healthy subjects. Moreover, this study suggests that mainly CAD acts as a key factor on serum and salivary vitamin C and antioxidant levels through a pathway mediated by the CRP. This pilot study is promising and demands further studies with a larger sample and longitudinal observation in saliva, serum, and gingival crevicular fluid in order to better understand the role of vitamin C levels during periodontitis.

## Figures and Tables

**Table 1:** Individual characteristics and biochemical parameters of recruited subjects.

	Controls (*N* = 36)	Periodontiti (*N* = 36)	CAD (*N* = 35)	Periodontitis + CAD (*N* = 36)
Age (years)	54 (51; 56)	55 (51; 57)	54 (48; 57)	55 (50; 56)
Gender (male/female)	17/19	18/18	19/17	17/19
Education level				
Primary school, *n* (%)	13 (36.1)	12 (33.3)	11 (31.4)	13 (36.1)
High school, *n* (%)	12 (33.3)	14 (38.9)	13 (37.1)	13 (36.1)
College/university, *n* (%)	11 (30.5)	10 (27.8)	11 (31.4)	10 (27.8)
Body mass index (kg/m^2^)	25.6 (21.9; 27.7)	25.1 (22.8; 26.7)	25.9 (21.8; 28.4)	25.5 (21.7; 27.1)
Fasting glucose (mg/dL)	93.5 (87.7; 97.9)	94.4 (84.3; 106.2)	92.6 (87.9; 109.1)	93.1 (88.7; 111.2)
Current smokers, *n* (%)	3 (8.3)	3 (8.3)	2 (5.7)	3 (8.3)
Comorbidities				
Diabetes, *n* (%)		2 (14.2) **	3 (8.6) **	3 (8.3) **
Previous CVD				
Atrial fibrillation, *n* (%)			7 (20) **^,§§^	10 (27.8) **^,§§^
Angina pectoris, *n* (%)			16 (45.7) **^,§§^	17 (48.6) **^,§§^
Stroke, *n* (%)			7 (20) **,^§§^	9 (25.7) **^,§§^
Heart failure, n (%)			9 (25.7) **^,§§^	10 (27.8) **^,§§^
Drug treatment of CVD				
Antihypertensive, *n* (%)			13 (37.1) **^,§§^	14 (38.9) **^,§§^
Statins, *n* (%)			9 (25.7) **^,§§^	10 (27.8) **^,§§^
Low-dose aspirin, *n* (%)			9 (25.7) **^,§§^	11 (30.5) **^,§§^
Beta blockers, *n* (%)			10 (28.5) **^,§§^	12 (33.3) **^,§§^
hs-CRP (mg/L)	2.8 (2.2; 3.1)	3.7 (2.9; 4.2) *	6.4 (5.1; 6.9) **	6.8 (5.9; 7.7) **^,§§,#^
Total cholesterol (mg/dL)	174 (131; 189)	178 (152; 194)	181 (155; 197) *^,§§^	180 (149; 200)
Triglycerides (mg/dL)	130 (111; 144)	131 (57; 151)	134 (109; 157) *^,§§^	136 (117; 161)
Retinol (µmol/L)	2.12 (1.75; 2.47)	2.08 (1.89; 2.09)	2.05 (1.81; 2.07) *^,§§^	2.04 (1.78; 2.15) *^,§§^
α-carotene (µmol/L)	0.081 (0.051;0.123)	0.075 (0.041; 0.101)	0.073 (0.051; 0.123) *^,§§^	0.071 (0.049; 0.112) *^,§§^
β-carotene (µmol/L)	0.321 (0.22; 0.367)	0.317 (0.209; 0.361)	0.311 (0.278; 0.302) *^,§§^	0.309 (0.214; 0.355) *^,§§^
β-cryptoxanthin (µmol/L)	0.066 (0.043; 0.78)	0.059 (0.033; 0.71)	0.051 (0.029; 0.82) *^,§§^	0.048 (0.039; 0.64) *^,§§^
γ-tocopherol (µmol/L)	2.66 (2.35; 2.99)	2.63 (2.21; 2.73)	2.59 (2.12; 2.85) *^,§§^	2.57 (2.04; 2.78) *^,§§^
Lutein (µmol/L)	0.16 (0.11; 0.26)	0.14 (0.1; 0.19)	0.12 (0.08; 0.18) *^,§§^	0.11 (0.09; 0.21) *^,§§^
Zeaxanthin (µmol/L)	0.037 (0.024; 0.045)	0.034 (0.018; 0.039)	0.029 (0.021; 0.027) *^,§§^	0.031 (0.021; 0.042) *^,§§^
Lycopene (µmol/L)	0.36 (0.21; 0.42)	0.35 (0.22; 0.41)	0.32 (0.21; 0.39) *^,§§^	0.31 (0.19; 0.36) *

Data are expressed as median (25th and 75th percentiles) or number with percentage. * *p* < 0.001 and ** *p* < 0.001 significant differences vs. healthy subjects calculated by the Mann–Whitney test. ^§§^ *p* < 0.001 significant differences vs. periodontitis patients calculated by the Mann–Whitney test. ^#^ *p* < 0.008 significant differences vs. CAD patients calculated by the Mann–Whitney test. CAD, ischemic heart disease; CVD, cardiovascular disease; hs-CRP, C-reactive protein.

**Table 2:** Clinical dental variables of recruited subjects.

	Controls (*N* = 36)	Periodontitis (*N* = 36)	CAD (*N* = 35)	Periodontitis + CAD (*N* = 36)
*N* of teeth	26 (24; 28)	19 (17; 20) **	23 (20; 24) **^,§§^	18 (13; 20) **^,##^
CAL (mm)	1.1 (0.8; 1.3)	4 (3.5; 4.2) **	2.1 (1.7; 2.4) **^,§§^	4.1 (3.6; 4.8) **^,##^
CAL 4–5 mm (% sites)		38.7 (36.2; 43.4) **		42.2 (38.9; 48.7) **^,##^
CAL ≥6 mm (% sites)		20.2 (16.8; 21.7) **		18.2 (16.4; 24.2) **^,##^
PD (mm)	1.4 (1.1; 1.8)	4.5 (4.1; 5.2) **	2 (1.9; 2.3) **^,§§^	4.1 (3.8; 4.7) **^,##^
PD 4–5 mm (% sites)		42.1 (40.1; 46.4) **		44.8 (41.5; 51.1) **^,##^
PD ≥6 mm (% sites)		22.3 (17.9; 23.1) **		23.9 (21.6; 27.6) **^,§§,##^
BOP (%)	8.8 (6.1; 10.9)	47.1 (45.1; 48.9) **	8.7 (5.2; 9.2) **^,§§^	45.7 (44.6; 56.2) **^,§§,##^
PI (%)	6.9 (5.3; 10.8)	34.9 (33.3; 36.1) **	12.9 (12.1; 13.4) **^,§§^	34.3 (31.2; 35.1) **^,##^

Data are expressed as median (25th and 75th percentile). ** *p* < 0.001 significant differences vs. control subjects calculated by the Mann–Whitney test. ^§§^ *p* < 0.001 significant differences vs. periodontitis patients calculated by the Mann–Whitney test. ^##^ *p* < 0.001 significant differences vs. CAD patients calculated by the Mann–Whitney test. CAL, clinical attachment level; PD, probing pocket depth; BOP, bleeding on probing; PI, plaque index.

**Table 3:** Uni- and multivariate linear regression model for serum and salivary vitamin C levels in all enrolled subjects.

	Univariate			Multivariate		
Variable	B	95% CI	*p*	B	95% CI	*p*
**Serum Vitamin C levels**						
CAD	−0.378	−0.222; 0.578	<0.001	−0.069	−0.341; 0.498	0.644
Periodontitis	−0.223	−0.016; 0.404	0.034	−0.141	−0.039; 0.389	0.112
hs-CRP	−0.119	−0.075; 0.137	<0.001	−0.112	0.065; 0.149	<0.001
Age (years)	0.078	0.29; 0.004	0.081	0.039	−0.148; 0.187	0.436
Female gender	−0.149	−0.041; 0.327	0.149	−0.178	−0.112; 0.344	0.078
Triglycerides	−0.066	−0.199; 0.078	0.209	0.074	−0.167; 0.366	0.141
**Salivary Vitamin C levels**						
CAD	−0.236	0.134; 0.41	<0.001	−0.029	−0.433; 0.312	0.655
Periodontitis	−0.064	−0.087; 0.214	0.387	0.005	−0.151; 0.184	0.972
hs-CRP	−0.078	0.038; 0.132	<0.001	0.077	0.061; 0.146	<0.001
Age (years)	0.041	−0.041; 0.012	0.207	0.012	−0.029; 0.036	0.786
Female gender	−0.038	−0.114; 0.226	0.419	0.079	−0.047; 0.239	0.178
Triglycerides	0.079	−0.178; 0.006	0.039	−0.714	−0.058; 0.241	0.028
Serum vitamin C	−0.149	−0.031; 0.378	0.079	−0.029	−0.223; 0.154	0.599

Age was included as a continuous variable. For periodontitis and CAD, controls served as reference. For gender, males served as reference. For education, primary school served as a reference.

## Supplementary Materials

The following are available online at https://www.mdpi.com/2072-6643/11/12/2956/s1: Table S1. STROBE Statement—checklist of items that should be included in reports of observational studies.
